# GEDS: A Gene Expression Display Server for mRNAs, miRNAs and Proteins

**DOI:** 10.3390/cells8070675

**Published:** 2019-07-03

**Authors:** Mengxuan Xia, Chun-Jie Liu, Qiong Zhang, An-Yuan Guo

**Affiliations:** Department of Bioinformatics and Systems Biology, Hubei Bioinformatics and Molecular Imaging Key Laboratory, Key Laboratory of Molecular Biophysics of the Ministry of Education, College of Life Science and Technology, Huazhong University of Science and Technology, Hubei, Wuhan 430074, China

**Keywords:** miRNA, mRNA, protein, gene expression, visualization

## Abstract

High-throughput technologies generate a tremendous amount of expression data on mRNA, miRNA and protein levels. Mining and visualizing the large amount of expression data requires sophisticated computational skills. An easy to use and user-friendly web-server for the visualization of gene expression profiles could greatly facilitate data exploration and hypothesis generation for biologists. Here, we curated and normalized the gene expression data on mRNA, miRNA and protein levels in 23,315, 9009 and 9244 samples, respectively, from 40 tissues (The Cancer Genome Atlas (TCGA) and Genotype-Tissue Expression (GETx)) and 1594 cell lines (Cancer Cell Line Encyclopedia (CCLE) and MD Anderson Cell Lines Project (MCLP)). Then, we constructed the Gene Expression Display Server (GEDS), a web-based tool for quantification, comparison and visualization of gene expression data. GEDS integrates multiscale expression data and provides multiple types of figures and tables to satisfy several kinds of user requirements. The comprehensive expression profiles plotted in the one-stop GEDS platform greatly facilitate experimental biologists utilizing big data for better experimental design and analysis. GEDS is freely available online.

## 1. Introduction

The quantification, comparison and visualization of gene expression from mRNA, miRNA and protein levels are key steps in making novel discoveries from different samples. High-throughput technologies such as RNA-Seq and reverse phase protein array (RPPA) have emerged as powerful methods for the quantitative measurement of RNAs and proteins [1,2]. In recent years, The Cancer Genome Atlas (TCGA) [3] and Genotype-Tissue Expression (GTEx) [4] projects produced RNA-Seq and RPPA data for tens of thousands of tumorous and normal tissues. Projects such as the Cancer Cell Line Encyclopedia (CCLE) [5] and MD Anderson Cell Lines Project (MCLP) [6] generated a variety of cancer cell-line RNA-Seq and RPPA protein data. These publicly available expression data provide unprecedented opportunities to better understand the genetic basis of cancers and tissues. Currently, GSCALite [7], cBioPortal [8], Expression Atlas [9] and GEPIA [10] have provided many useful visualization and analysis tools for gene expression analysis. Among the numerous useful features provided by these tools, Expression Atlas distinguishes itself by providing multi-species expression data. GSCALite, cBioPortal and GEPIA mainly focus on the mRNA expression of human cancers and normal tissues. Although these tools are exceptionally valuable and widely used, some additional quick analyses requested by experimental biologists are not adequately addressed by those existing tools; for example, choosing specific cell lines for further study according to preliminary results, analyzing gene expression from RNA level and protein level as well as phosphorylated protein across multiple tissues and cell lines, and visualizing miRNA expression between tumor and normal tissues. Based on these required analyses, we developed the Gene Expression Display Server (GEDS, , http://bioinfo.life.hust.edu.cn/web/GEDS/), a comprehensive resource for searching and visualizing expression data of genes, miRNAs and proteins to complement the existing tools.

## 2. Data Sources and Implementation

### 2.1. Data Curation

**Figure 1:**
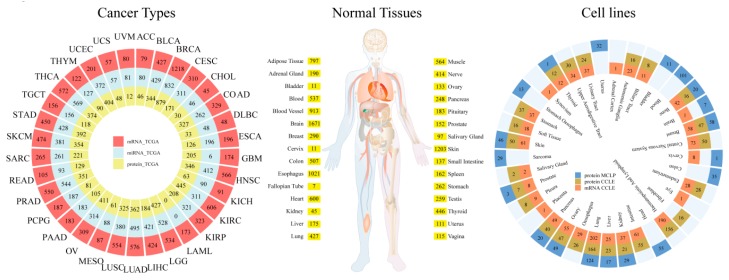
The data summary of the Gene Expression Display Server (GEDS). GEDS collected expression data of cancer types, normal tissues, and cell lines from The Cancer Genome Atlas (TCGA), Genotype-Tissue Expression (GTEx), Cancer Cell Line Encyclopedia (CCLE) and MD Anderson Cell Lines Project (MCLP).

We obtained the normalized mRNA, miRNA, and protein expression data from four prevalent data resources (TCGA, GTEx, CCLE and MCLP). These expression data were categorized into cancer types, normal tissues and cell lines, as shown in Figure 1. (1) mRNA expression data are from 9744 tumor and 727 normal samples across 33 cancer types of TCGA, 11,688 samples across 30 normal tissues of GTEx and 1156 cancer cell lines across 30 tissues of CCLE. (2) Protein and phosphorylated protein level expression data for more than 300 cancer-related proteins include 7672 tumor samples across 32 cancer types from TCGA, 651 cancer cell lines across 20 tissues from MCLP and 899 cancer cell lines across 24 tissues from CCLE. (3) miRNA expression data include 8389 tumor and 620 normal samples across 32 cancer types from TCGA. We used in-house scripts to process these datasets. Briefly, for TCGA datasets, according to cancer types and TCGA patient barcodes, we calculated the expression quantile of each gene, protein and miRNA in tumor and normal samples, respectively. We separated GTEx data by tissues and calculated the expression quantile of each gene across normal tissues. Based on the documents of cell line lineages, we classified CCLE and MCLP cell lines into tissues and calculated the expression quantile of each molecule in all cell lines of a tissue. These multi-source and multi-platform expression data from different projects integrated in GEDS were normalized, and we removed batch effects across tissues and cell lines by their maintainers. Therefore, the expression values from different sources (projects) cannot be compared directly, but we can order and compare the expressions from the same project. For example, we can compare the tissue expression among samples from TCGA, but cannot compare expressions between TCGA and GTEx data.

### 2.2. User Interface

The user interface and back-end of GEDS were built with Shiny. Programing language R and R-package plotly (https://plot.ly/r/) were used to generate figures and tables. All analysis results were presented on a web page and could be downloaded in PNG format. GEDS provides a user-friendly interface that enables the user to intuitively determine the expression of mRNA, protein and miRNA in tissues and cell lines. Users can search for the expression of mRNA, protein and miRNA in different panels. (1) In the mRNA panel, when users input a list of gene symbols (e.g., TP53) or aliases (e.g., p53) and click the search button, GEDS will present the expression profile of each gene with a boxplot. (2) In the miRNA panel, users can input a list of miRNA official IDs (e.g., miR-9-5p) or short IDs (e.g., miR-9), and it will display the expression of each miRNA in TCGA cancer types. (3) In the protein panel, users can select a specific protein or phosphorylated protein status. The protein expression quantified by RPPA is presented as a boxplot. (4) Publication-ready figures and normalized expression data are provided and can be downloaded for further analysis.

## 3. Use Case

**Figure 2:**
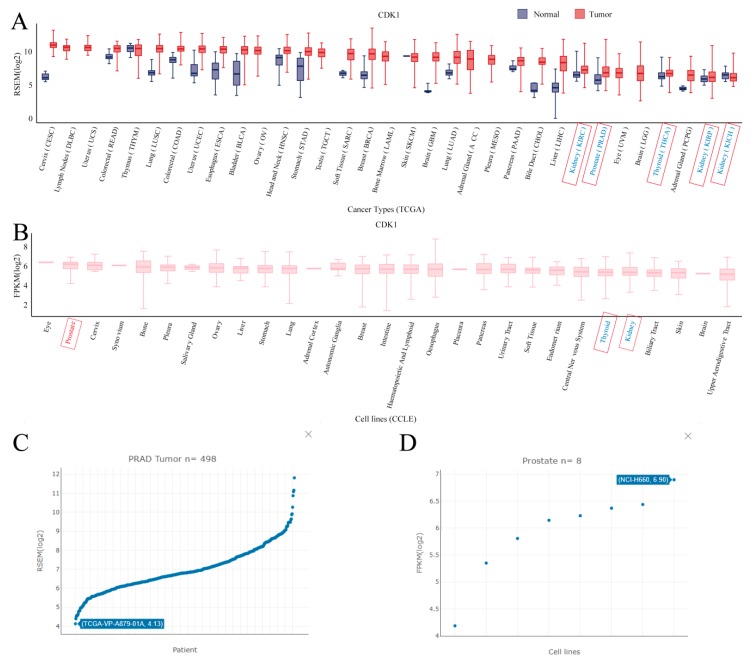
The use case of GEDS taking CDK1 as an example. The figure presents the results of CDK1 mRNA expression in the TCGA dataset (**A**) and CCLE dataset (**B**). Words in red and blue are the cancer types or tissues with high and low CDK1 expressions, respectively. The detailed expression of CDK1 in each prostate cancer sample (**C**) and cell line (**D**).

A previous study reported that gene expression patterns can be different between cancer cell lines and cancer patient samples in some type of cancers [11]. We considered CDK1 as an example to present gene expression differences between cell lines and tissues through GEDS. The mRNA level of CDK1 has higher expression in cervix cancers (CESC) and lower expression in cancer of the kidney renal papillary cell carcinoma (KIRP), kidney renal clear cell carcinoma (KIRC), kidney Chromophobe (KICH) and thyroid carcinoma (THCA) across the TCGA dataset (Figure 2A). Meanwhile, in the CCLE dataset, the mRNA level of CDK1 had a consistent expression in these tissues (Figure 2B). However, in prostate cancer, CDK1 showed a significant difference between TCGA (Rank 27) and CCLE (Rank 2), showing the different expression patterns between prostate cancer samples and cell lines. In the detailed expression of each prostate cancer sample (Figure 2C) and cell line (Figure 2D), NCI-H660 showed the highest expression of CDK1. This suggests that NCI-H660 may be the best cell line to analyze the difference between prostate cancer cell lines and prostate cancer tissue.

## 4. Discussion

We developed a highly useful web-server, GEDS, for gene expression quantification, comparison and visualization. GEDS is a time-saving and intuitive tool for unleashing the value of the larger amount of gene expression data, which enables experimental biologists without any computational programming skills to analyze large amounts of expression data, and thereby to choose an experimental system and test hypothesis. We anticipate that such a display server will be popular for the mainstream analysis of gene expression. GEDS will be continuously maintained and refined upon user feedbacks.
